# Identification of a Novel G2073A Mutation in 23S rRNA in Amphenicol-Selected Mutants of *Campylobacter jejuni*

**DOI:** 10.1371/journal.pone.0094503

**Published:** 2014-04-11

**Authors:** Licai Ma, Zhangqi Shen, Gaowa Naren, Hui Li, Xi Xia, Congming Wu, Jianzhong Shen, Qijing Zhang, Yang Wang

**Affiliations:** 1 Key Laboratory of Development and Evaluation of Chemical and Herbal Drugs for Animal Use, College of Veterinary Medicine, China Agricultural University, Beijing, People's Republic of China; 2 Department of Veterinary Microbiology and Preventive Medicine, College of Veterinary Medicine, Iowa State University, Ames, Iowa, United States of America; Institut National de la Recherche Agronomique, France

## Abstract

**Objectives:**

This study was conducted to examine the development and molecular mechanisms of amphenicol resistance in *Campylobacter jejuni* by using *in vitro* selection with chloramphenicol and florfenicol. The impact of the resistance development on growth rates was also determined using *in vitro* culture.

**Methods:**

Chloramphenicol and florfenicol were used as selection agents to perform *in vitro* stepwise selection. Mutants resistant to the selective agents were obtained from the selection process. The mutant strains were compared with the parent strain for changes in MICs and growth rates. The 23S rRNA gene and the L4 and L22 ribosomal protein genes in the mutant strains and the parent strain were amplified and sequenced to identify potential resistance-associated mutations.

**Results:**

*C. jejuni* strains that were highly resistant to chloramphenicol and florfenicol were obtained from *in vitro* selection. A novel G2073A mutation in all three copies of the 23S rRNA gene was identified in all the resistant mutants examined, which showed resistance to both chloramphenicol and florfenicol. In addition, all the mutants selected by chloramphenicol also exhibited the G74D modification in ribosomal protein L4, which was previously shown to confer a low-level erythromycin resistance in *Campylobacter* species. The mutants selected by florfenicol did not have the G74D mutation in L4. Notably, the amphenicol-resistant mutants also exhibited reduced susceptibility to erythromycin, suggesting that the selection resulted in cross resistance to macrolides.

**Conclusions:**

This study identifies a novel point mutation (G2073A) in 23S rRNA in amphenicol-selected mutants of *C. jejuni*. Development of amphenicol resistance in *Campylobacter* likely incurs a fitness cost as the mutant strains showed slower growth rates in antibiotic-free media.

## Introduction

*Campylobacter jejuni* is a leading bacterial cause of acute gastroenteritis in humans [1]. *Campylobacter* infection is also the most common antecedent to Guillain-Barré syndrome (GBS), manifested as symmetric ascending paralysis [2], [3]. Usually, *Campylobacter* infections are self-limiting; however, clinical antimicrobial treatment is warranted in patients with severe or long-lasting infections, or with compromised immune systems [4], [5]. However, antimicrobial resistance in *C. jejuni* has increased significantly over the past decades, compromising clinical treatments and presenting a major public health threat [6]–[9].

Chloramphenicol (CHL) and florfenicol (FFC) are members of the amphenicol family, which are highly effective against a wide variety of Gram-positive and Gram-negative bacteria. Amphenicols were once widely applied in both human and veterinary practice for the prevention and treatment of many bacterial infections. Florfenicol, the fluorinated derivative of chloramphenicol, has been licensed for the control of bacterial respiratory tract infections in several food production animals, including cattle and pigs [10]. Nowadays, the use of chloramphenicol is limited to a small number of life-threatening infections in humans because of its adverse effects, which include bone-marrow depression, aplastic anaemia, and acute leukaemia [11]–[13]. The use of chloramphenicol in food-producing animals was banned in many countries; however, it is still widely used in pets and non-food-producing animals [14]–[16]. Chloramphenicol binds directly to the peptidyltransferase centre on the 50S ribosomal subunit, preventing peptide bond formation [17]. Acetylation of the drug by chloramphenicol acetyltransferase, which does not mediate resistance to FFC, is the most frequently encountered mechanism of bacteria resistance to CHL [10]. Other mechanisms of CHL resistance include efflux systems such as *cmlA* [18], *floR* [19], *fexA* [20], *fexB* [21], *pexA* [22]; point mutations in domain V of the 23S rRNA [23]; and methylation of A2503 in the 23S rRNA gene of the large ribosomal subunit, which is catalysed by *cfr* methyltransferase [24].

A series of studies showed that CHL resistance was very common in *E. coli* (40.6–79%) [25], [26] and *Salmonella* (28.42%) [27], while chloramphenicol resistance in *Campylobacter* rarely occurs. No chloramphenicol-resistant isolates were detected in studies performed in the United States [28], Spain [29], Japan [30], and Iran [31]. The chloramphenicol resistance detection rates were 0.8% in Korea [32]. In contrast to the low resistance rates in other countries, detection of chloramphenicol-resistant *Campylobacter* was high (37.5%) in Brazil [33]. In China, the florfenicol and chloramphenicol resistance rates of *Campylobacter* isolated from chicken [34] reached up to 61.7 and 24.5%, respectively. Chloramphenicol resistance in *Campylobacte*r is mediated by chloramphenicol acetyltransferases [35]–[37]. To date, there have been no reports on FFC resistance mechanisms in *Campylobacter*. In this study, we examined the development of CHL and FFC resistance mechanisms in *C. jejuni* by *in vitro* selection and assessed the impact of the resistance on *Campylobacter* fitness.

## Materials and Methods

### Bacterial strains and culture conditions

The *C. jejuni* strain ATCC 33560, susceptible to CHL and FFC (MIC_CHL_ = 4 µg/mL and MIC_FFC_ = 2 µg/mL), was used as the parent strain for the selection studies with CHL and FFC. *C. jejuni* was cultured routinely on Mueller-Hinton agar (MHA, Sigma, St. Louis, MO) or in Mueller-Hinton broth (MHB, Sigma) for 24–48 hours at 42°C under microaerobic conditions (5% O_2_, 10% CO_2_, and 85% N_2_). If necessary, the MH media were supplemented with various concentrations of CHL or FFC. All strains were preserved in MH broth with 20% glycerol at −80°C.

### CHL & FFC susceptibility testing

The MICs against CHL and FFC were determined using the agar dilution method as recommended by the Clinical and Laboratory Standards Institute [38]. According to the recommended breakpoints of NARMS [39], isolates were considered resistant to CHL and FFC if their MICs were ≥32 and ≥8 µg/mL, respectively. CHL, FFC, erythromycin, azithromycin, spiramycin, and clindamycin were purchased from the China Institute of Veterinary Drug Control, Beijing. Phenylalanine-arginine-β-naphthylamide (PAβN, Sigma-Aldrich, St. Louis, MO), an efflux pump inhibitor, was also used in this study to identify whether the antibiotic efflux systems of *C. jejuni* played a role in the resistance. The concentration of PAβN used in this study was 30 µg/mL [40]. A control plate of MH agar containing PAβN of the same concentration was also included to assess the effect of PAβN on the growth of the isolates investigated. The experiments mentioned above were repeated three times.

### *In vitro* selection of CHL and FFC resistant strains

CHL- and FFC-resistant strains were selected *in vitro* with CHL and FFC as the selective agents, respectively. Briefly, the parent strain ATCC 33560 was first cultured on antimicrobial-free MHA media. For first round of selection, cultures of the parent strain were inoculated onto MHA media containing CHL or FFC at 0.5-fold MICs, and incubated under microaerobic conditions at 42°C for 3–5 days. For subsequent stepwise selection, cultures were collected and transferred to MHA containing a 2-fold higher concentration of antibiotics than the concentration used in the preceding round of selection. The highest concentrations of selective agents are 16-fold and 64-fold MIC for CHL and FFC, respectively. Single colonies (n = 10) were picked and tested at each stepwise selection. MICs of resistant clones against CHL and FFC and erythromycin, azithromycin, spiramycin, and clindamycin were determined accordingly.

### Growth rates of the parent and mutant strains in MH broth

To compare the growth kinetics of selected resistant strains (C3 and F5) with the susceptible parent strain, a fresh culture of each strain was separately and equally inoculated into antimicrobial-free MH broth at an initial cell density of OD_600_ = 0.05. The cultures were incubated at 42°C under microaerobic conditions, and then aliquots of the samples were collected at 0, 4, 8, 12, 24, and 36 hours post-inoculation for OD_600_ determination. Three independent experiments were performed.

### Sequence analysis of genes encoding 23S rRNA, L4 ribosomal protein, and L22 ribosomal protein

With respect to the 23S rRNA gene, we used the specific primer pair 23S-F and 23S-R to amplify all three copies of the 23S rRNA gene to detect potential CHL or FFC resistance-associated mutations (Table 1). To detect which specific copy of the gene contained the mutation, PCRs were performed to amplify each copy of the domain V of 23S rRNA gene. The three pairs of operon-specific PCR primers (FI/CJ copy-R; FII/CJ copy-R; and FIII/CJ copy-R) are listed in Table 1 [41]. The amplification reactions were performed with premixed LA Taq (TaKaRa Co. Ltd., Dalian, China), and the cycling parameters were 95°C for 5 min, followed by 30 cycles of 95°C for 30 sec for denaturation, 55°C for 30 sec for annealing, and 68°C for 5 min for extension. The PCR cycles were followed by a final extension period of 15 min at 68°C. The PCR products were purified by using TIANGEN DNA midi purification kit (TIANGEN, Beijing, China) and subsequently sequenced.

**Table 1:** Primers used in this article.

Primers	Sequence (5′-3′)	References
23S-F	5′-AGCTACTAAGAGCGAATGGT-3′	this study
23S-R	5′-AAAGATAAGCCAAACGCTCT-3′	this study
FI	5′-CCCTAAGTCAAGCCTTTCAATCC-3′	[40]
FII	5′-CGTTATAGATACGCTTAGCGGTTATG-3′	[40]
FIII	5′-CATCGAGCAAGAGTTTATGCAAGC-3′	[40]
CJ copy-R	5′-CTACCCACCAGACATTGTCCCAC-3′	[40]
L4-F	5′-GTAGTTAAAGGTGCAGTACCA-3′	[40]
L4-R	5′-GCGAAGTTTGAATAACTACG-3′	[40]
L22-F	5′-GAATTTGCTCCAACACGC-3′	[40]
L22-R	5′-ACCATCTTGATTCCCAGTTTC-3′	[40]

The sequences of the L4 and L22 ribosomal protein genes from the resistant clones were analysed in comparison with the parental strain using primers as previously described [42]. The PCR cycling conditions for the L4 and L22 genes were as follows: initial denaturation at 95°C for 5 min, followed by 30 cycles of 95°C for 30 sec, 55°C for 30 sec, 72°C for 45 sec, with a final extension at 72°C for 5 min. The PCR products were purified and sequenced for point mutations.

## Results

### Development of CHL and FFC resistance in C. jejuni by *in vitro* selection

Through independent stepwise *in vitro* selection experiments with CHL or FFC as the selective agents, we acquired *C. jejuni* strains of different resistance levels to the antibiotics. The selected resistant strains are listed in Table **2**. Three successive generations of CHL-resistant strains (MIC_C1_ = 32 µg/mL; MIC_C2_ = 64 µg/mL; and MIC_C3_ = 128 µg/mL) and five successive generations of FFC-resistant strains (MIC_F1_ = 16 µg/mL; MIC_F2_ = 32 µg/mL; MIC_F3_ = 64 µg/mL; MIC_F4_ = 128 µg/mL; and MIC_F5_ = 256 µg/mL) were chosen for further analysis. MICs of these strains were determined with or without PAβN in comparison with the parent strain ATCC 33560. As shown in Table 2, the selected mutants showed ≥8-fold increases in the MICs of CHL and FFC compared to ATCC33560. Addition of PAβN in the assay made little differences in the MICs (no change or 2-fold decrease; Table 2), and the PAβN alone did not affect the growth of the isolates investigated. As shown in Table 2, all CHL-resistant strains and high-level FFC-resistant strains (including F3, F4, and F5) showed decreased susceptibility to the both selective agents. Interestingly, the MICs of erythromycin against mutant strains C3 and F5 increased eight-fold (from 1 to 8 µg/mL), and the MICs of azithromycin increased four-fold (from 0.25 to 1 µg/mL), but were still lower than the resistance breakpoints (≥16 and ≥8 µg/mL, respectively), suggesting that selection with amphenicol led to a low-level cross-resistance to macrolides. However, the MICs of spiramycin and clindamycin only increased two-fold (data not shown).

**Table 2:** Target mutations identified in CHL- and FFC-resistant mutants.

	MIC (µg/mL)^b^									
Strains a	CHL	CHL+PAβN	FFC	FFC+PAβN	Erythromicin	Azithromycin	Mutation in the 23S rRNA gene c	Change in protein L4	Change in protein L22	Selective Agents e
ATCC33560	4	4	2	2	1	0.25	-d	-	-	NA
C1	32	32	16	16	8	1	-	G74D	-	CHL
C2	64	32	32	16	8	1	G2073A	G74D	-	CHL
C3	128	64	64	64	8	1	G2073A	G74D	-	CHL
F1	8	4	16	8	2	0.5	-	-	-	FFC
F2	16	8	32	32	8	1	G2073A	-	-	FFC
F3	32	8	64	32	8	1	G2073A	-	-	FFC
F4	64	16	128	64	8	1	G2073A	-	-	FFC
F5	64	32	256	128	8	1	G2073A	-	-	FFC

a C1-C3 were selected with CHL, while F1-F5 were selected with FFC.

b Determined by the standard agar dilution method. ND, not determined.

c The G2073A mutation was present in three copies of the domain V region of 23s rRNA gene.

d no mutation detected.

e CHL: Chloramphenicol; FFC: Florfenicol; NA: not applicable.

### Molecular mechanism of Chloramphenicol and Florfenicol resistance

- Analysis of domain V of the 23S rRNA gene
-
  - PCRs were performed with specific primers 23S-F and 23S-R (Table **1**) to identify the potential mutations in domain V of the 23S rRNA gene in the selected resistant *C. jejuni* strains. Sequence analysis showed that a G-to-A single nucleotide change was detected at position 2073 of 23S rRNA (corresponding to position 2057 in the 23S rRNA gene of *Escherichia coli*) of all the mutants examined (Table 2). Further studies were performed with operon-specific primer pairs (FI/Cj copy R, FII/Cj copy R, and FIII/Cj copy R) to amplify and analyse the three copies of the 23S rRNA gene in the mutant strain. The results indicated that these mutants exhibited the G2073A mutation in all three copies of the gene (Table **2**). When analysing the process of resistance development, we did not found the mutation in any of the three copies of the 23S rRNA gene in the FFC-selected clones with MIC_FFC_ ≤16 µg/mL and the CHL-selected clones with MIC_CHL_ ≤32 µg/mL.
- Sequence analysis of L4 and L22 ribosomal protein genes

- The selected mutant strains and the parent strain were examined for alterations in the L4 and L22 ribosomal protein genes. The G74D change, which was previously reported to confer low-level resistance to erythromycin in *Campylobacter* [42]–[44], was detected in the L4 protein in all the CHL-selected mutants (MIC_CHL_≥16 µg/mL), but was absent in the FFC-selected strains, regardless of the resistance levels FFC (Table 2). No mutations were detected in the L22 ribosomal protein gene in either CHL-selected or FFC-selected mutants (Table 2).

### Growth kinetics of the resistant strains

**Figure 1:**
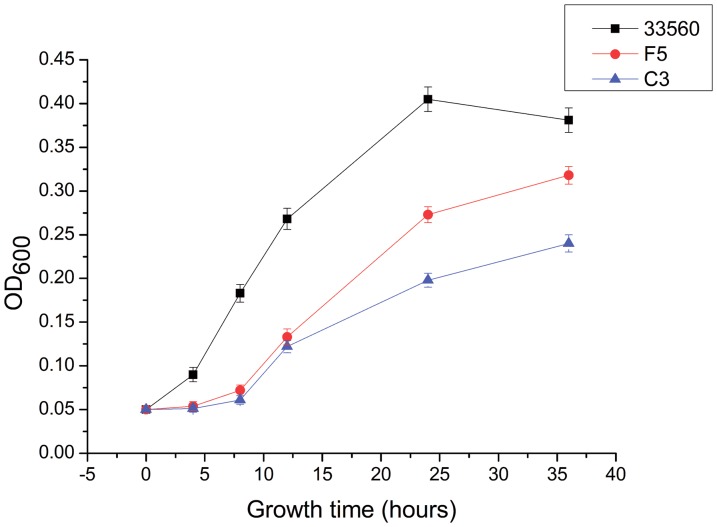
Growth kinetics of the wild-type strain and mutant strains of *C. jejuni* in MH broth. The OD_600_ were measured at 0, 4, 8, 12, 24 and 36 hours post-inoculation. Two mutant strains (C3 and F5) and the parent strain ATCC 33560 were included in the experiment. The experiment was repeated three times, and the results were shown as mean of three independent experiments.

Two mutant strains (C3 and F5) and the parent strain were separately inoculated into antimicrobial-free MH broth and tested for growth rates. For the first 8 hours of incubation post-inoculation, the two mutant strains showed little growth (Figure 1). According to the OD values, the C3 and F5 strains showed apparent differences in growth kinetics between the wild-type parent strain and the mutants, suggesting that the C3 and F5 mutants might have impaired fitness.

## Discussion

*C. jejuni* has become increasingly resistant to antimicrobials, which poses a significant threat to human health. There were numerous studies on antimicrobial resistance of *Campylobacter* species [45]-[47], but very few focused on the mechanism of CHL and FFC resistance [36]. In this study, *C. jejuni* strain ATCC 33560, which usually serves as a quality-control strain for the antimicrobial susceptibility testing of *Campylobacter* spp. [38], [48], was used as the parent strain to select for CHL- or FFC-resistant *C. jejuni in vitro* [49].

In this study, some unique features were revealed concerning the development of CHL and FFC resistance in *C. jejuni*. Firstly, irrespective of the selective agent, the resistant clones obtained *in vitro* exhibited dual resistance to both agents (Table **2**). Similar cross-resistance was previously reported in *E. coli* and *Salmonella* [50], [51], which usually emerged under FFC selection. Combined resistance to CHL and FFC might be conferred by specific resistance genes, such as *floR* [19], *fexA* [20], *fexB* [21], *pexA* [22] and *cfr* [24]. Secondly, both the CHL- (MIC_CHL_≥64 µg/mL) and FFC-selected (MIC_FFC_≥32 µg/mL) resistant strains contained the G2073A mutation in three copies of the 23S rRNA gene (Table **2**), which is the first reported mutation associated with amphenicol resistance in *Campylobacter* spp. The G2057A mutation of the *E. coli* 23S rRNA gene (corresponding to position 2073 in the 23S rRNA gene of *Campylobacter* spp.) was previously reported to confer resistance to chloramphenicol and intermediate-level resistance to 14-nunbered-ring macrolides. Indeed, the CHL- and FFC-selected *C. jejuni* mutants carrying the G2073A mutation also showed elevated MICs to erythromycin (8 µg/mL), consistent with the finding in *E. coli* that the G2057A conferred cross resistance to chloramphenicol and erythromycin [23]. Interestingly in propionibacteria [32], *Mycoplasma hominis* [33], and *Francisella tularensis* [31], the same G2057A mutation was associated with erythromycin resistance, but not with resistance to 16-membered macrolides or chloramphenicol. It was postulated that the G2057 mutation in the 23S rRNA gene might lead to conformational changes in the binding sites of CHL and 14-membered macrolides, attenuating the affinity of these antimicrobials to the ribosome [52].

The G74D modification was found in the L4 ribosomal protein of CHL-selected resistant strains (MIC_CHL_≥16 µg/mL), while the modification was absent in FFC-selected mutants, suggesting that the structural differences between CHL and FFC might contribute to the difference in the mutant selection process. FFC is a synthetic derivative of CHL with a fluoro group substitution at position C_3_ and the replacement of a nitro group (-NO_2_) by a sulfomethyl group (-SO_2_CH_3_) [53]. It is also possible that there are differences in the binding sites for CHL and FFC in ribosomal protein L4, or differences in the mode of action between CHL and FFC. The G74D mutation is located in a conserved region of the L4 ribosomal protein, which is considered as the main anchoring site of this ribosomal protein to 23S rRNA [54]. Several previous reports also identified the G74D mutation in ribosomal protein L4 of *Campylobacter* spp. [33], [42], [55], and this mutation, by itself, usually conferred low-level resistance to erythromycin and contributed to higher-level resistance when combined with the efflux pump CmeABC [56] or a G57V mutation in L4 [43]. Furthermore, the result from the PAβN assay suggested that the efflux mechanism might not play a major role in resistance to CHL and FFC in the mutant strains that carried the G2073A mutation. Thus, the G2073A rRNA mutation alone may be sufficient to confer resistance to CHL and FFC in *C. jejuni* mutants. It was reported in *E. coli* that the G2057A change in combination with other mutations in the 23S rRNA gene (i.e., G2032A) conferred a higher level of chloramphenicol resistance [57].

Several studies investigated the fitness of antibiotic-resistant *Campylobacter* spp. with mutation-associated resistance [58]–[60]. In the study of Luo *et al*. [58], they reported that fluoroquinolone-resistant *Campylobacter* showed an enhanced fitness *in vivo*. However, macrolide-resistant *Campylobacter* demonstrated a severe defect in both *in vitro* and *in vivo* fitness [60]. These finding indicate that different target mutations have varied impact on *Campylobacter* fitness. The results from the growth experiment in this study (Figure 1) demonstrated that the G2073A alteration in the 23S rRNA gene had a negative effect on the growth rates of the mutant strains, which suggests that development of amphenicol resistance incurs a fitness cost in *Campylobacter*. It appeared that the CHL-selected strains exhibited the most significant fitness disadvantage (Figure **1**).

In conclusion, we discovered a novel G2073A mutation in the 23S rRNA gene of *C. jejuni* that is associated with amphenicol resistance. This mutation was identified by *in vitro* selection using CHL or FFC. Notably, this G2073A mutation was also associated with reduced susceptibility to erythromicin, suggesting that it confers cross resistance to both amphenicols and macrolides. Considering that the amphenicol resistance rates in *Campylobacter* are rising in certain countries, it will be interesting to determine if this resistance-associated mutation is naturally present in clinical isolates and if the amphenicol-resistant mutants are able to continue to persist in the absence of antibiotic selection pressure.
